# Community based trial of home blood pressure monitoring with nurse-led telephone support in patients with stroke or transient ischaemic attack recently discharged from hospital

**DOI:** 10.1186/1745-6215-9-15

**Published:** 2008-03-19

**Authors:** Sally Kerry, Hugh Markus, Teck Khong, Reena Doshi, Rachel Conroy, Pippa Oakeshott

**Affiliations:** 1 Community Health Sciences, St George's, University of London, London, UK; 2 Cardiac and Vascular Sciences, St George's, University of London, London, UK; 3 Basic Medical Sciences, St George's, University of London, London, UK

## Abstract

**Background:**

High blood pressure in patients with stroke increases the risk of recurrence but management in the community is often inadequate. Home blood pressure monitoring may increase patients' involvement in their care, increase compliance, and reduce the need for patients to attend their General Practitioner if blood pressure is adequately controlled. However the value of home monitoring to improve blood pressure control is unclear. In particular its use has not been evaluated in stroke patients in whom neurological and cognitive ability may present unique challenges.

**Design:**

Community based randomised trial with follow up after 12 months.

*Participants*: 360 patients admitted to three South London Stroke units with stroke or transient ischaemic attack within the past 9 months will be recruited from the wards or outpatients and randomly allocated into two groups. All patients will be visited by the specialist nurse at home at baseline when she will measure their blood pressure and administer a questionnaire. These procedures will be repeated at 12 months follow up by another researcher blind as to whether the patient is in intervention or control group.

*Intervention*: Intervention patients will be given a validated home blood pressure monitor and support from the specialist nurse. Control patients will continue with usual care (blood pressure monitoring by their practice).

Main outcome measures in both groups after 12 months:

1. Change in systolic blood pressure.

2. Cost effectiveness: Incremental cost of the intervention to the National Health Service and incremental cost per quality adjusted life year gained.

**Trial registration:**

Clinical Trials.gov registration NCT00514800

## Background

Poorly controlled blood pressure (BP) in people with stroke or transient ischaemic attack (TIA) increases the risk of recurrence but is difficult to manage in the community. Our recent meta-analysis provides some evidence that home monitoring of BP is associated with better BP control [1]. If true, this could be important for stroke prevention. However there was considerable heterogeneity between trials and the current value of self monitoring remains uncertain [2,3]. Many studies showing reductions in BP were underpowered, had inadequate length of follow up or were done many years ago [1,4]. Reduction of antihypertensive treatment based on home BP instead of clinic BP may lead to less intensive drug treatment and worse control [5], and there is an urgent need for an economic evaluation of home BP monitoring [6]. No trials have been conducted in people with stroke [4].

Home BP monitoring could benefit people with stroke both by improving BP control thus reducing the risk of recurrent stroke, and by enabling them to be more involved in treatment decisions [2,7]. It has been shown to improve compliance with treatment and to remove white coat syndrome [5]. McManus et al recently found practice based self monitoring was well received by patients with no apparent increase in anxiety or cost [4]. However attending a general practice may be difficult or inconvenient for many people with stroke. Home BP monitoring can reduce the number of visits to the surgery [8]. But in people with stroke it may present particular challenges due to the neurological and cognitive disability which may affect both ability to successfully use the equipment and compliance.

Following our meta-analysis of home monitoring [1], we have just completed a community based study of nurse led BP management [9]. As in our proposed trial, this study included 25% of patients of African origin who are known to be at increased risk of stroke [10,11]. In addition our ongoing experience in GP outreach hypertension clinics suggests that the use of home BP monitoring is increasing and is feasible in patients with stroke or TIA. Self monitoring should include regular professional supervision and check of the patient's measuring technique [2], clear treatment goals adjusted for lower home readings, and a willingness to intensify drug treatment [3,6]. Combined with nurse led support, the introduction of home BP monitoring is a realistic and pragmatic intervention for patients in general practice which is where most BP management takes place. Before home BP monitoring becomes widespread in the UK, there is an opportunity for a robust trial to see if it is a feasible, effective and cost effective option for stroke patients living in the community [3].

### Questions to be answered

a. Does home monitoring of BP and patient held BP targets with support from a specialist nurse lower systolic BP after 12 months compared with usual care?

b. Is home blood pressure monitoring cost effective?

c. What is the effect of home BP monitoring on quality of life, anxiety, number of prescribed anti hypertensive drugs and number of medication changes?

d. How does disability affect the success of home monitoring in people with stroke?

## Methods/Design

### Recruitment

Patients will be recruited from three South London stroke units: St George's, St Helier and Mayday hospitals.

#### Identification of patients

The research nurse will ask one of the stroke doctors or nurses which patients on the stroke/TIA ward or stroke/TIA clinic might be suitable to be approached about the study. Information sheets about the trial and a card with inclusion/exclusion criteria will be available on the wards and in the clinics.

##### Inclusion criteria

• Patient has had a stroke or TIA within the last 9 months.

• Patient is hypertensive defined as on antihypertensive treatment for raised BP, or BP>140/85 mmHg when measured more than a week after a stroke/TIA.

##### Exclusion criteria

• Severe illness or another major illness likely to dominate the pattern of care eg advanced cancer.

• Already using a home BP monitor.

• Non-English speaking.

• Severe cognitive impairment defined as Abbreviated Mental Test Score (AMTS) <7.

• Known secondary hypertension.

#### Consent procedure

The research nurse will introduce herself and ask the patient (and carer if present) if they would consider helping in a research study of home BP monitoring in people with stroke or TIA. If they are interested she will give them an information sheet to read, show them a BP monitor, briefly explain the study and answer any questions.

If the patient is willing to take part, she will give them a consent form to sign.

For patients who wish to discuss the study with their GP or other people first, she will give them a consent form and stamped addressed envelope to complete and post back if they wish to participate.

#### Information to be obtained at the time of consent

Date of birth, gender, contact details and details of GP, date of most recent stroke or TIA.

In addition the research nurse will classify the degree of stroke related disability using the modified Rankin score and confirm that participants are hypertensive (on antihypertensive therapy or BP>140/85 mmHg). These data will be entered on the preliminary data collection sheet. Age, sex and Rankin score will be used in the randomisation procedure. The nurse will carry out an AMTS assessment to check eligibility for the study.

#### Medical record search

Patients signing the consent form agree for the researchers to have access to their hospital and GP medical records. When possible, clinical details, including imaging and stroke sub-typing and risk factors profile will be extracted from the hospital records and/or the local hospital stroke register where available, and entered on the trial data collection sheet.

### Randomisation

Patients who consent will be stratified by age, sex and Rankin score and randomly allocated into two groups by the principal investigator (Sally Kerry) using a computer programme. The allocation for each patient will be put in a sealed envelope which will be opened by the specialist nurse after completion of the baseline assessment at home.

### Information for the patient's GP (all patients)

The research team will write to the patient's GP informing them of the patient's participation in the study explaining that the BP target for home monitoring (<130/80 mm Hg) is lower than that for clinic readings. As with hospital letters, we will request that the letter is scanned into the patient's computerised GP medical records and filed in their paper notes. In addition the GP will be sent a laminated A4 copy of the NICE/BHS ACD 2006 Hypertension Guidelines [7], and a brief outline of the study's aims and design. The letter will be sent just prior to the baseline home visit. We anticipate patients will come from about 100 different GP practices.

### Baseline assessment at home

The research nurse will telephone the patient to arrange a convenient time to visit. The visit will be done within 2 months of discharge from the ward or recruitment in the clinic. She will answer the patient's questions, confirm they are still willing to participate and carry out the baseline assessment.

#### Baseline clinical measurements

**BP **and pulse rate will be measured with an automatic machine (Omron 705 CP sphygmomanometer with printout) after the participant has been sitting upright for at least 5 minutes. The appropriate cuff size will be used (13 by 32, or 16 by 42 cm if arm circumference is ≥ 33 cm). Three readings will be taken 1 minute apart. The first will be discarded and the mean of the last two used in the analysis.

If BP is consistently over 170/105 mm Hg, including when repeated at the end of the home visit, and the patient is relaxed and has been taking their medication regularly, she will advise them to see their GP in the next 3 days. (If BP is consistently above 210/115 mmHg, she will advise them to see the GP immediately). If tablets have been missed she will emphasise the importance of taking medication regularly to prevent another stroke/TIA, and ask them to see their GP in the next two weeks.

**Height **will be measured when possible without shoes using a height rule to the nearest 0.5 cm.

**Weight **will be measured to the nearest 0.5 kg with manual Seca 761 scales.

These will be entered on the data collection sheet.

#### Structured Questionnaire

The nurse will administer a questionnaire. This will include:

1. Self-assigned ethnicity.

2. Lifestyle: smoking, alcohol, exercise, dietary salt, and fruit and vegetable intake.

3. Medical history: previous stroke or TIA, diabetes, atrial fibrillation, cardiovascular disease.

4. Current drug treatment and knowledge of target BP.

5. Validated instruments: Quality of life (EQ-5D) [12], Level of anxiety (FEAR 4 item scale) [13].

### Treatment allocation

After completing the baseline assessment the nurse will open the sealed envelope to see if the patient is in the intervention or control group.

### Control group

These patients will continue with usual care -BP monitoring by their practice. The research nurse will telephone them at 1, 6 and 9 months and encourage healthy diet and exercise.

### Intervention group

Patients in the intervention group will receive all of the following:

#### Home BP monitor

The nurse will give the patient (and/or carer) a validated BP monitor Omron M6 with appropriate size cuff and teach the patient or carer how to take their BP using a standardised method and taking 3 readings each time [14]. She will give them a specially prepared booklet "Blood Pressure Record Booklet". This explains the method of BP measurement, target BP and actions to be taken if BP is above target, and also has tables in which to record BP. The nurse will advise the participant to measure their BP every morning and evening under standardised conditions for a week (omitting the first 24 hours readings) and then weekly on the same day each week and record readings in the booklet.

#### Target BP

The nurse will explain that home BP target is <130/80 mm Hg [7] (less if diabetic) and that their GP may not know this as this is less than clinic targets. *A sticker *with target BP and the nurse's telephone number will be put on the BP monitor. Instructions on the record card will advise the patient what to do if readings taken on 3 different occasions are consistently raised. First telephone the specialist nurse for advice in case they are taking their BP wrongly or the machine is faulty. If appropriate, the nurse may visit them again within a week to check this. Otherwise for BP consistently 131/81–169/104 mmHg make a routine appointment with GP. For BP 170/105–209/114 see GP within 3 days. For BP over 210/115 mmHg see GP or attend A+E immediately.

#### Telephone support from specialist nurse

The nurse will encourage patients to ring her mobile in working hours if they have any queries. This is particularly important in the first week of using the home BP monitor. If she is unavailable they should leave a message so she can ring them back.

#### Follow up home visit after a month and continued telephone support

After a month the nurse will revisit to check that patients are taking their BP correctly and record any problems patients are having with either using the equipment or understanding the results. For those with BP consistently above target she will advise the patient to see their GP taking their BP booklet with them. The booklet gives the GP a record of the home BP readings with the target home BP of <130/80 mmHg clearly shown. She will also give them a note to give the GP suggesting s/he considers changing antihypertensive treatment, and a copy of the 2006 joint NICE/BHS ACD guidelines [7]. Following this second visit she will provide telephone support 2 weekly and a further visit if appropriate until target BP is reached, with further telephone calls at 6 and 9 months.

### Assessment of outcome after 6 and 12 months in intervention and control groups

#### 6 month assessment at home

An independent researcher unaware of whether the patient is in the intervention or control group will assess the patient at home. She will explain that she should not know which group the patient is in so at both assessment visits the patient should put away their BP monitor if they have one and avoid telling her whether or not they are monitoring their BP. At this assessment s/he will just record BP (using the Omron 705CP automatic sphygmomanometer with printout) according to the standardised method set out previously.

#### Final 12 month assessment at home

At 12 months s/he will measure BP (according to the standardised method) and weight before administering the final questionnaire. This should reduce any effect of possible unblinding. As at baseline, the questionnaire will include:

1. Lifestyle: smoking, alcohol, exercise, dietary salt, and fruit and vegetable intake.

2. Medical history in the past year: previous stroke or TIA, diabetes, atrial fibrillation, cardiovascular disease.

3. Current drug treatment and knowledge of target BP.

4. Validated instruments: Quality of life (EQ-5D) [11], Level of anxiety (FEAR 4 item scale) [13].

To capture any impact of the intervention on other areas of the health service [15], the questionnaire will also ask about health service use. We will ask how many times in the last 12 months the participant has: a) seen their GP b) seen a practice/community nurse c) had an outpatient appointment d) had a hospital stay. For at least 10% of patients we will cross-validate their questionnaire data on usage of the health service in the past year using practice records.

### Outcome measures

#### Primary outcome

Change in systolic BP after 12 months.

#### Secondary outcomes

Change in systolic BP after 6 months; change in diastolic BP after 6 and 12 months, number of prescribed antihypertensive drugs; number of changes in prescribed antihypertensive drugs; change from baseline in scores for EQ-5D, FEAR questionnaire, and knowledge of target BP.

#### Cost effectiveness

Incremental cost of the intervention to the NHS [15] and incremental cost per quality adjusted life year gained (QALY). To be consistent with other studies we will follow the methods of the reference case as advocated by the NICE [16]. To calculate QALYs, life expectancy will be estimated on the basis of the measured risk factors; and improvements in quality of life will be estimated using the EQ-5D at baseline and twelve months [17,18]. We expect the main cost of the intervention to the health service will be the nurse time. The research nurse will complete a timesheet distinguishing between time spent on the intervention from time spent on research elements of the project. Resource use will be costed using standard NHS sources [19-21].

### Statistical analysis and sample size

The primary analysis will compare change in systolic blood pressure over the year between the intervention and control groups using a t test. Secondary analysis will compare secondary outcomes in intervention and control groups but also investigate effect of disability and cognition on change in systolic blood pressure and nurse's record of difficulties with home monitoring in the intervention group. Characteristics of patients recruited will be compared with those on the Stroke Register not taking part to assess the generalisability of the results to stroke patients. A sample size of 322 will be able to detect a difference of 5 mmHg [1] in change at 12 months between the intervention and control group with 80% power using a 5% significance level assuming the SD is 16 mmHg [9]. Allowing for a 10% loss to follow up [12] we will need to recruit 360 patients.

## Conclusion

If home blood pressure monitoring is shown to be feasible, effective and cost effective, encouraging people with stroke to use home BP monitoring could improve BP control and reduce risk of recurrence. It will also assist GPs and practice nurses in the management of blood pressure in patients with stroke.

## Abbreviations

ACD Hypertension Guidelines – A:Angiotensin converting enzyme inhibitor, C:Calcium channel blocker, D:Thiazide-type Diuretic; AMTS – Abbreviated Mental Test Score; BHS – British Hypertension Society; BP – Blood Pressure; GP – General Practitioner; NICE – National Institute for Health and Clinical Excellence; PO – Pippa Oakeshott; QALY – Quality Adjusted Life Year; SD – Standard Deviation; SK – Sally Kerry; TIA – Transient Ischaemic Attack

## Competing interests

The author(s) declare that they have no competing interests.

## Authors' contributions

SK and PO conceived the idea for the study and participated in the design of the study. SK was responsible for the statistical analysis plan and sample size calculations. HM and TK also contributed to the design of the study. RD and RC drafted the manuscript for submission to Trials and were involved in compiling patient information and data collection packs. All the authors have read and approved the final manuscript.
